# Automatic parameter selection for electron ptychography via Bayesian optimization

**DOI:** 10.1038/s41598-022-16041-5

**Published:** 2022-07-19

**Authors:** Michael C. Cao, Zhen Chen, Yi Jiang, Yimo Han

**Affiliations:** 1 Department of Materials Science and NanoEngineering, Rice University, Houston, TX 77005 USA; 2 School of Materials Science and Engineering, Tsinghua University, Beijing, 100084 China; 3 Advanced Photon Source, Argonne National Laboratory, Lemont, IL 60439 USA

**Keywords:** Transmission electron microscopy, Characterization and analytical techniques

## Abstract

Electron ptychography provides new opportunities to resolve atomic structures with deep sub-angstrom spatial resolution and to study electron-beam sensitive materials with high dose efficiency. In practice, obtaining accurate ptychography images requires simultaneously optimizing multiple parameters that are often selected based on trial-and-error, resulting in low-throughput experiments and preventing wider adoption. Here, we develop an automatic parameter selection framework to circumvent this problem using Bayesian optimization with Gaussian processes. With minimal prior knowledge, the workflow efficiently produces ptychographic reconstructions that are superior to those processed by experienced experts. The method also facilitates better experimental designs by exploring optimized experimental parameters from simulated data.

## Introduction

Ptychography is a computational imaging method that has gained great interests in the electron microscopy community^1–4^. The technique was first proposed by Hoppe in 1969^5^ and re-invigorated in recent years with the developments of fast electron detectors^6–11^ that can rapidly collect thousands of diffraction patterns per second. Various iterative reconstruction algorithms have been developed to retrieve the scattering potentials of the sample and the wave function of the illumination from intensity measurements^12–14^. It has been demonstrated that electron ptychography can break the Abbe diffraction limit of imaging systems^15^ and set a new world record in spatial resolution (0.39 Å) in atomically thin two-dimensional (2D) materials^3^. As one of the phase-contrast imaging techniques, electron ptychography also has high dose efficiency for low-dose imaging ranging from low-dimensional nanomaterials^16,17^ to biological specimens^18,19^. An even more critical breakthrough is that electron ptychography can inversely solve the long-standing problem of multiple scattering in thick (> 20 nm) samples and enables a lattice-vibration-limited resolution (0.2 Å)^4^, as well as three-dimensional depth sectioning^4,20^.

Despite its great success in achieving record-breaking resolution, ptychography remains a niche technique in electron microscopy due to many practical challenges in both experimental setup and data analysis. In particular, there exist many types of parameters that significantly influence image quality and need to be carefully selected for different data or applications. For example, physical parameters that describe processes such as noise generation, partial coherence, and probe vibration can be modeled in an iterative ptychographic reconstruction, which essentially solves a non-convex optimization problem. Choosing appropriate parameters to account for these practical errors is paramount to achieving solutions that are close to the real object. Other parameters, including the number of iterations, update step size, and initial probe, also influence reconstructions by controlling the convergence process. For simplicity, in the work, we categorize all parameters described above as *reconstruction parameters*. In addition, *experimental parameters*, such as scan step size, probe defocus, and camera length also need to be determined before measurement and often limit the best image quality of a given data. Due to virtually infinite possibilities and complex trade-offs between various parameters, it is practically impossible to design and optimize ptychography experiments by searching the entire parameter space. In practical applications^3,4,16,17^, scientists often select parameters manually based on their experiences with the sample or instrument. This can potentially introduce biases to scientific conclusions drawn from the results. Although a few key parameters were systematically studied in previous literature^18,21,22^, exploring multiple parameters greatly reduces the overall throughput and creates a high barrier for general researchers to adopt the technique.

Here we present a general framework for fully automatic parameter tuning in electron ptychography by leveraging Bayesian optimization (BO) with Gaussian processes^23^—a popular strategy for global optimization of unknown functions. Using experimental ptychography data and state-of-the-art reconstruction algorithms, we demonstrated that our approach can automatically produce high-resolution images after exploring only 1% of the discretized reconstruction parameter space. We also optimized experimental parameters for ultra-low electron dose levels, providing insights for more robust experimental designs that further to enhance ptychography’s usability. Instead of relying on human intuition and judgment, automatic parameter selection promotes objective and reproducible protocols, paving the way for fully autonomous experiments and data processing for ptychography applications.

## Results

### Bayesian optimization with Gaussian process

Bayesian optimization with Gaussian process is frequently used to find global maxima and minima of a black-box function that is unknown and expensive to evaluate. The technique has been used in a wide variety of applications in machine learning^24,25^, Monte Carlo simulation^26^, and autonomous controls in microscopy experiments^27–29^. In general, BO consists of three steps: (1) compute a surrogate function that models the true objective function based on sampled points, (2) determine the next point(s) to be sampled based on an acquisition function, (3) evaluate the objective function at the corresponding points. The surrogate function is described by kernel functions, which affect the periodicity, smoothness, and length scales of the objective function. It also predicts values and their standard deviations at unsampled points, which is used by the acquisition function to balance finding the extrema (exploitation) or reducing the uncertainty in the surrogate (exploration). In contrast, direct search methods such as Mesh Adaptive Direct Search^30^ or Nelder-Mead^31^ that do not use a surrogate are more likely to get trapped in local minima^32^.

**Figure 1:**
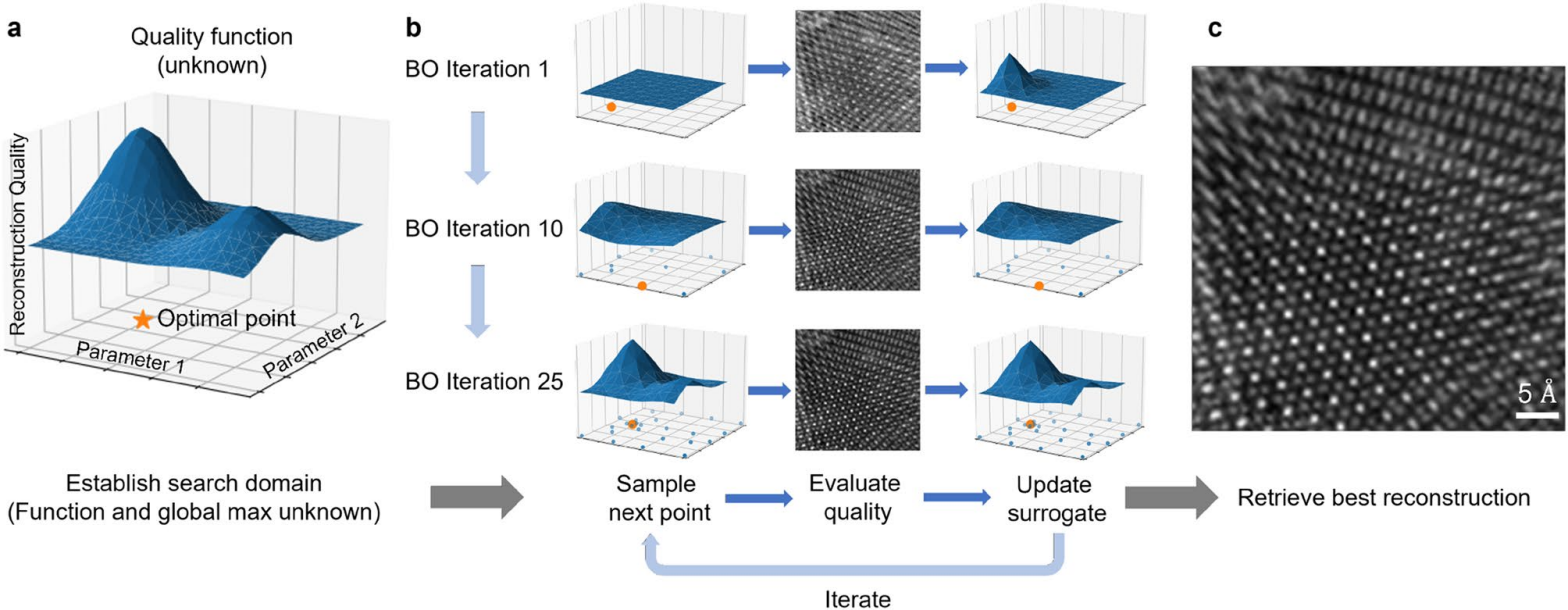
Schematic of automatic reconstruction tuning with Bayesian optimization. (**a**) The process aims to find the best ptychographic reconstruction by optimizing an unknown quality function that is data-dependent in general. (**b**) Bayesian optimization loop strategically determines the next point (indicated in orange) to sample, performs ptychographic reconstruction, and then updates the surrogate model based on the image quality. As the number of iterations increases, the surrogate model becomes closer to the true quality function and more points around the optimum are exploited. (**c**) The image with the best quality during BO is retrieved as the final reconstruction.

In ptychography, we utilize BO to optimize an objective function that evaluates reconstruction quality and varies for each dataset. Figure 1 illustrates the complete workflow. The initial set of ptychography reconstructions are generated based on randomly chosen parameters. Based on these reconstructions, Gaussian process models a surrogate function, and candidate points thereafter are chosen according to the acquisition function. The following reconstructions are performed with these parameters, the quality is measured, and the surrogate and acquisition functions are updated. The updated acquisition function then suggests the next candidate set and the process is iterated (Fig. 1b). After sufficient iterations, the set of parameters that generate the highest quality reconstruction is determined (Fig. 1c).

### Automatic reconstruction parameter tuning

To demonstrate BO as an efficient framework for automatic selection of reconstruction parameters, we apply the approach to an experimental dataset of bilayer MoSe$_2$/WS$_2$ that is publicly available from ref.^16^. Ptychographic reconstructions were carried out using the least square maximum likelihood (LSQ-ML) algorithm^33^ implemented in the PtychoShelves package^34^, which incorporates many advanced techniques such as mixed-states ptychography^35^, position correction^33^, variable probe correction^36^, batch update^33^, and multislice ptychography^4,37^. These features play crucial roles in previous works that successfully achieved dose-efficient and large field of view (FOV) imaging^16,38^ as well as deep sub-angstrom spatial resolution of thick crystalline materials^4^. Because different parameters have different computational costs per iteration, we perform time-limited reconstructions that terminate after reaching a time threshold specified by the user. This provides more practical comparisons by balancing the trade-offs of parameters such as the number of probe modes and batch size. Assuming strong phase approximation, which generally works well for 2D materials, the parameter space is discretized and consists of eight common parameters with 4800 possible combinations. Detailed descriptions of each reconstruction parameter are provided in the “Methods” section and Supplementary Table 1.

**Figure 2:**
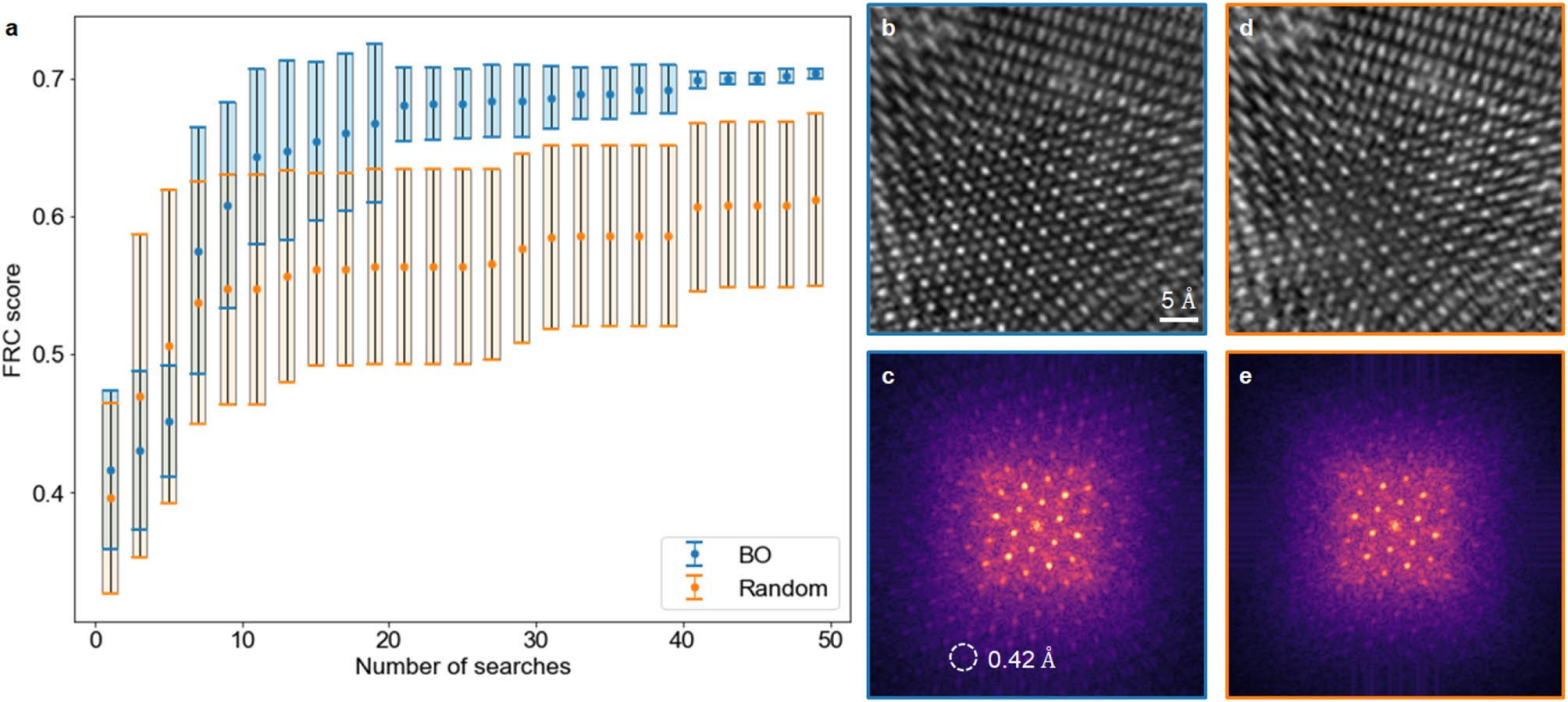
Performance of Bayesian optimization versus a random search. (**a**) Plot of FRC score behavior over the number of parameter searches using BO versus a random search strategy. In 10 trials, BO consistently outperforms random search with higher FRC score and lower uncertainties as the number of parameter searches increases. (**b**,**d**) Best reconstruction for one trial after 50 parameter searches using (**b**) Bayesian optimization and (**d**) random search. (**c**,**e**) Diffractograms of reconstructions in (**b**) and (**d**), respectively.

Fourier ring correlation (FRC) analysis^39^ was used as a quantitative metric to evaluate the quality of ptychographic reconstructions. Without the “ground truth” for experimental data, FRC analysis measures the similarity between two independent reconstructions and is often used to estimate “spatial resolution” in phase retrieval problems^40^ or cryogenic electron microscopy reconstructions^41^. Our automatic parameter tuning workflow aims to maximize the area under the normalized FRC curve, ranging from 0 to 1 with 1 corresponding to identical images. The process starts by trying 5 initial random sets of reconstruction parameters, then leverages BO to search for the next point that is most likely to produce better reconstruction quality, and stops after exploring 50 points in total—only 1% of the entire parameter space. A fully random parameter selection strategy was also investigated as comparison. Each sampling strategy was carried out 10 times (with different initial starts) and the averaged best FRC scores and their standard deviation after each search are shown in Fig. 2a. It is obvious that BO can consistently achieve higher FRC scores than random sampling, even if it starts with worse averaged FRC scores. The frequency of specific reconstruction parameter values also demonstrates that BO tends to sample more points around the optimal parameters. For example, for all 3-minute reconstructions, the percentage of position correction used in BO and random sampling are 71.2% and 48.4%, respectively. Selected reconstructions from BO and random sampling are shown in Fig. 2b,d, respectively. The reconstructed atoms from BO are visibly sharper than the ones from random sampling, which agrees with the evaluation based on FRC and their diffractograms (Fourier intensity) (Fig. 2c,e). It is worth noting that the diffraction spot that corresponds to an Abbe resolution of 0.42 Å is visible in the reconstruction with optimized parameters, surpassing the resolution (0.69 Å) reported in our previous work^16^.

**Figure 3:**
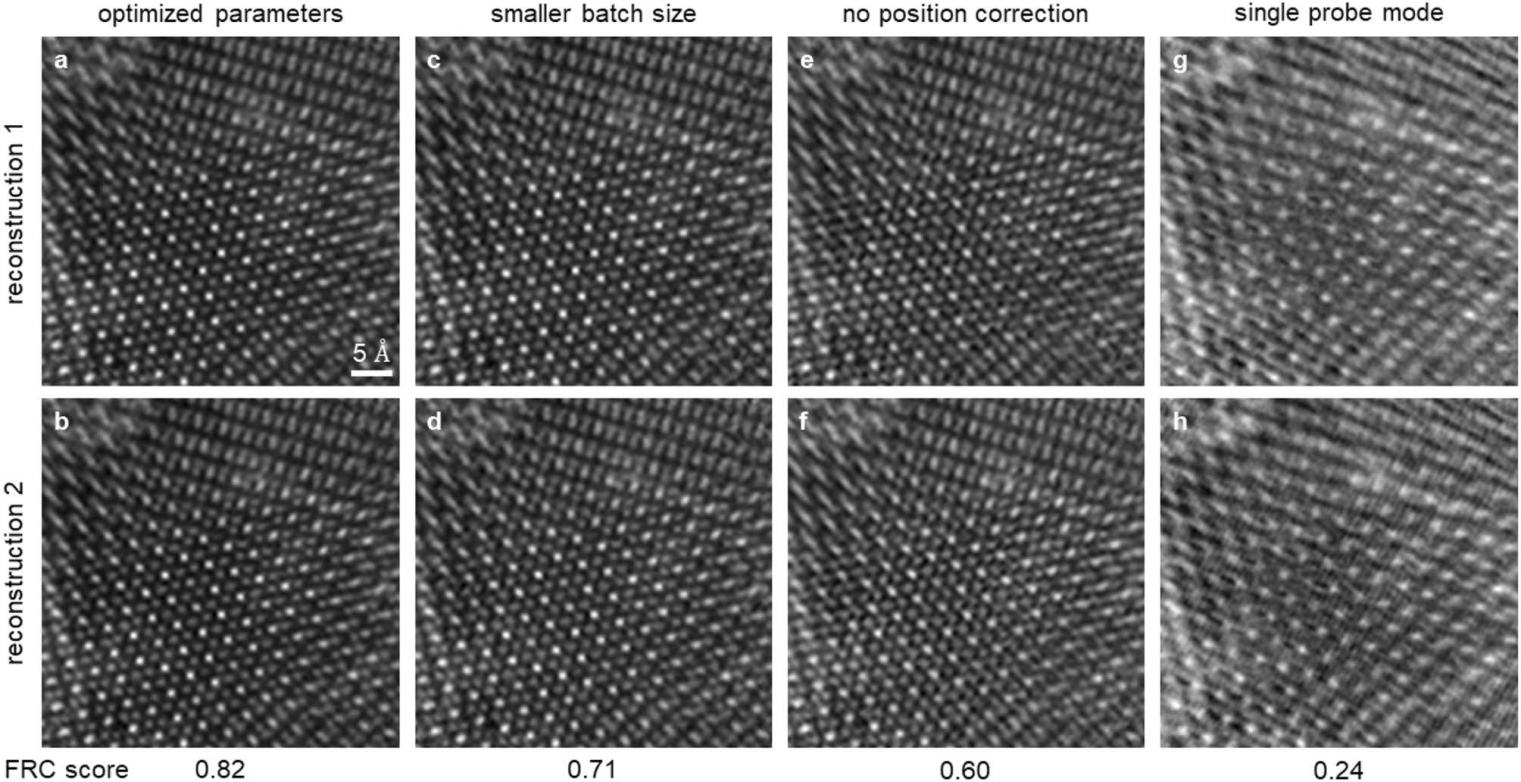
Optimized ptychographic reconstructions of bilayer MoSe$_2$/WS$_2$ compared with sub-optimal parameters. Two datasets that cover the same scan area were reconstructed independently for 5 min using the LSQ-ML technique. (**a**,**b**) Reconstructed phase with the parameters optimized by Bayesian optimization, including 7 mixed-state probe modes, a batch size of 300, and scan position correction. (**c**–**h**) Reconstructions where one of the optimal parameters is changed. All sub-optimal parameter combinations decrease the reconstruction quality to varying degrees. (**c**,**d**) Reconstructions with a batch size of 60, which increases the time per iteration, and reduces the total number of iterations. (**e**,**f**) Reconstructions without position correction. (**g**,**h**) Reconstructions with a single probe mode.

Figure 3 illustrates the importance of reconstruction parameter tuning by showing 5-min reconstructions. The best results (Fig. 3a,b) found by BO used 7 mixed-states probe modes, a sparse batch size of 300, a Gaussian noise model, and position correction. These parameters agree well with the choice made by experienced scientists who are familiar with the algorithm and the data^16^. For comparison, a smaller batch size of 60 produces reconstructions (Fig. 3c,d) with broader atoms and a slightly lower FRC score than the optimal results. Moreover, reconstructions with no position correction and only a single probe mode are shown in Fig. 3e–h, respectively. These two features often have the largest effects on electron ptychography data as they correct major experimental errors such as partial coherence, beam vibration, sample drift, and scan noises. Without them, the reconstructed images have significantly worse quality and larger inconsistencies that are reflected in their FRC scores. The complete list of reconstruction parameters used for Fig. 3 is summarized in Supplementary Table 2.

**Figure 4:**
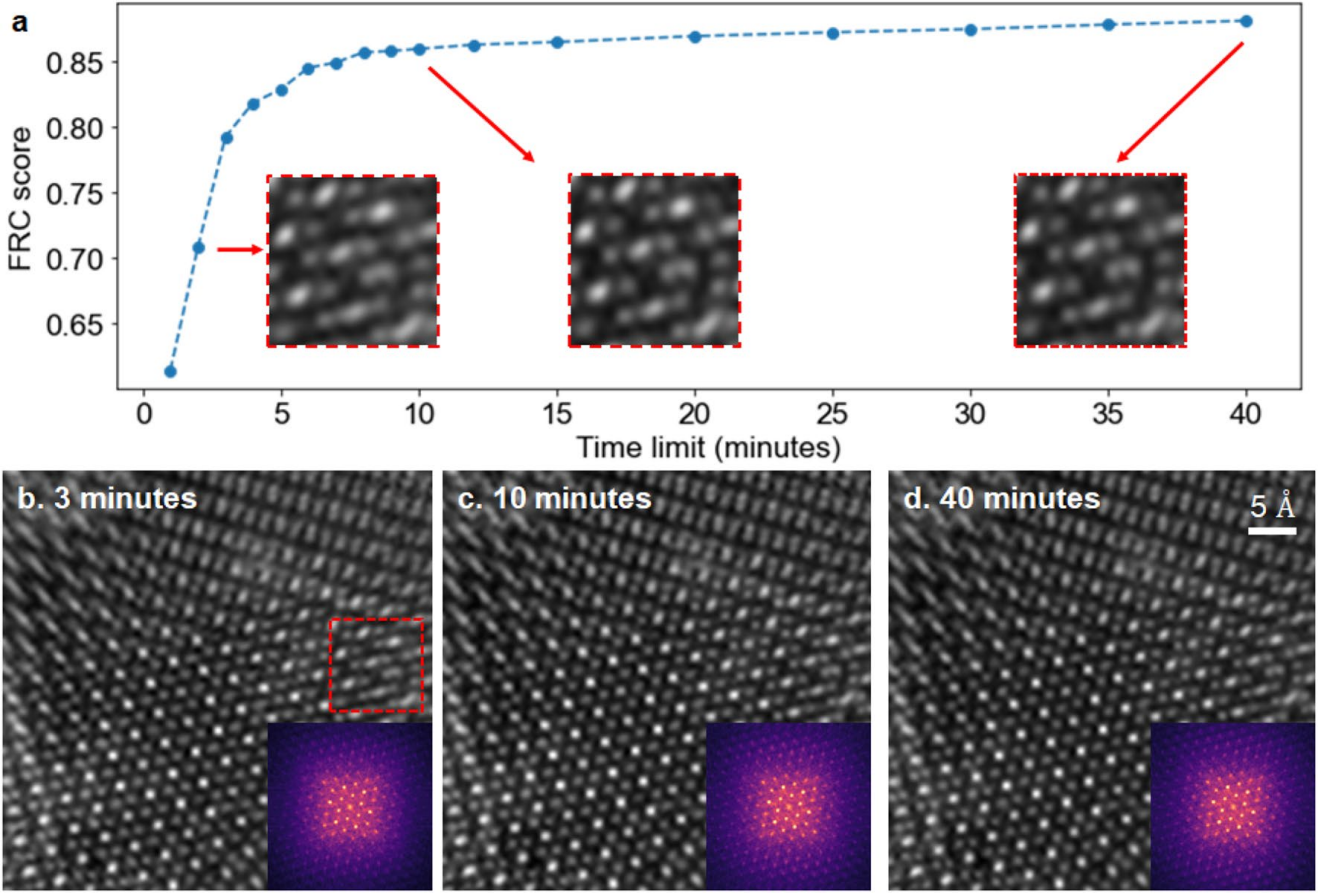
Ptychographic reconstructions of bilayer MoSe$_2$/WS$_2$ at different time limits. The best reconstruction parameters at each time limit were automatically optimized by BO. (**a**) Best FRC scores vs. reconstruction time. (**b**–**d**) Best reconstructions and their diffractograms (insets) after 3, 10, and 40 min, respectively. The zoom-ins in (**a**) shows that reconstructed atoms become more resolved as time increases.

Using the efficient and automatic parameter tuning enabled by BO, one can gain deeper understandings of optimal reconstruction parameters and systematically study how they change with time. As shown in Fig. 4, for the experimental dataset of MoSe$_2$/WS$_2$, the phase of the object converges to the bilayer structures within 10 min, as the probe modes quickly become more physical shapes. As time further increases, the FRC scores continue to improve, mainly thanks to scan position correction that often requires more iterations to refine large drifts or global position errors. The plots for individual reconstruction parameters are provided in Supplementary Fig. 1. For all time limits, BO indicates that the best results are obtained with a large number of probe modes, position correction, and a Gaussian noise model. It also suggests that no probe variation correction is needed—this is within our expectations since the scan FOV is relatively small (5 nm × 5 nm). In the early reconstruction stage (< 25 min), the sparse batch selection scheme leads to higher FRC scores since the algorithm has a faster initial convergence rate. On the other hand, given enough time (number of iterations), the algorithm benefits more from the compact batch selection scheme that is known to have slower convergence but is more robust to noise^33^. We further compared the reconstructed atomic distances with the structure model and confirmed that the compact batch indeed produces more accurate results than the sparse batch at longer time limits.

### Optimization of experimental parameters for low-dose ptychography

The dose of the illumination beam plays a crucial role in electron microscopy. For example, a high electron dose can damage the sample structure by energetic electrons, especially for radiation-sensitive samples, such as batteries, metal-organic frameworks, or biological materials^42,43^. In contrast, low dose mode results in noisy diffraction patterns, reducing spatial resolution or even introducing additional artifacts in ptychographic reconstructions. Therefore, it is critical to explore optimal imaging conditions at the allowed dose of illumination.

In general, experimental conditions, such as scan step size and probe size, often determine the best quality one can achieve after ptychographic reconstruction. The optimal experimental parameters should balance various physical factors such as the signal-to-noise ratio (SNR) of diffraction patterns, scanning probe overlap, and the sampling requirement in the detector plane^44,45^. Due to the complex trade-offs between different factors, it is generally challenging, even for human experts, to determine the optimal parameters that maximize the reconstruction quality in different experiments. For instance, at a fixed electron dose, a small scan step size leads to a large number of diffraction patterns with low electron counts collected in the detector. Increasing scan step size could improve the SNR but reduce the spatial overlap between adjacent probes. Although larger probe defocuses could provide better overlap in real space, it requires higher sampling (more pixels) in the detector plane, which again lowers the SNR since the averaged electron count per pixel is decreased.

**Figure 5:**
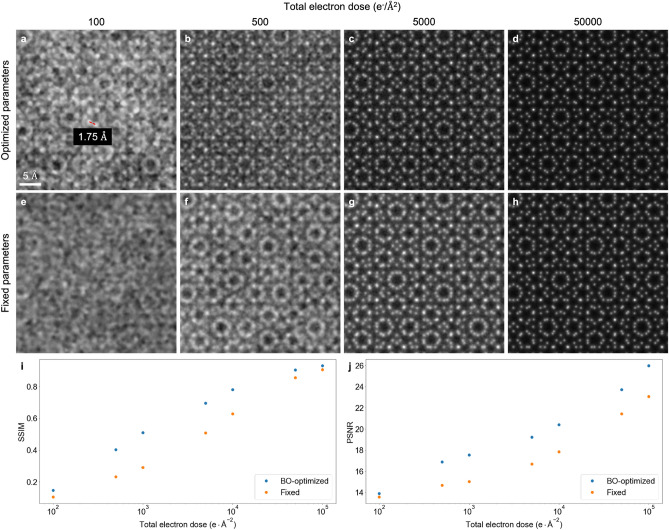
Ptychographic reconstructions of simulated bilayer MoS$_2$ at different total electron doses with an optimized set of experimental parameters compared with an expert-chosen set. (**a**–**d**) Phase of the reconstructed objects using experimental parameters that are optimized by Bayesian optimization. (**e**–**h**) Reconstructions using a fixed set of experimental parameters that are similar to ref.^16^. (**i**,**j**) SSIM and PSNR of reconstructions at different dose levels, respectively.

Using Bayesian optimization with Gaussian processes, we performed a comprehensive and automatic parameter tuning to search for the optimal scan step size, aperture size, probe defocus, and detector size (Supplementary Table 3) at different electron dose levels. For each point in the 4D parameter space, we first simulated diffraction patterns using a twisted bilayer MoS$_2$ structure (Supplementary Fig. 2) as the test object and carried out ptychographic reconstructions using the LSQ-ML algorithm. With the ground truth available, BO directly maximizes the accuracy of reconstruction, which is quantified using the structural similarity index measure (SSIM)^46^. The metric explicitly calculates the difference between two images in terms of luminance, contrast, and structure, providing a general evaluation of reconstruction quality. The Gaussian width used in SSIM was set to 1.5. As illustrated in Supplementary Fig. 3, other widths and evaluation metrics such as peak signal-to-noise ratio (PSNR) have similar parameter spaces and optimal points. Figure 5a–d shows the best reconstructions after 800 points are explored at various dose levels from 100 to 50,000 e$^-$/Å$^2$. As references, reconstructions with a fixed set of experimental parameters (2 Å scan step size, 20 mrad aperture size, -55 nm defocus, 256 × 256 detector size), which are similar to the ones used in ref.^16^, are shown in Fig. 5e–h. For quantitative evaluations, the SSIM and PSNR of reconstructions are shown in Fig. 5i,j, respectively. The experimental parameters optimized by BO produce significantly better resolution and more accurate structures, especially at lower dose levels where the physical requirements for good reconstructions are more stringent. At high dose levels, the data have sufficient SNR and the reconstruction quality becomes less sensitive to experimental parameters.

**Figure 6:**
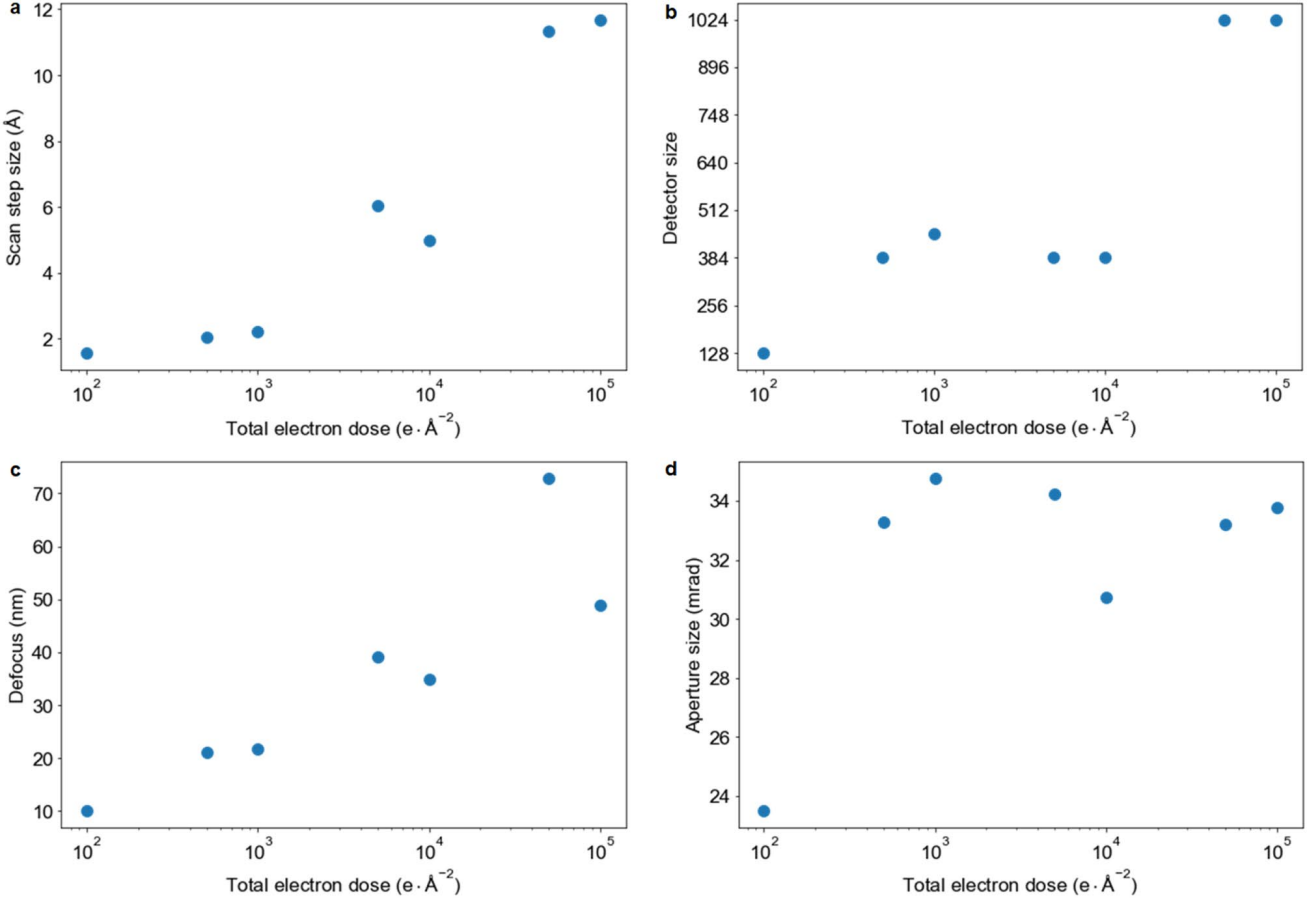
Optimal experimental parameters for electron ptychography. At each electron dose level, BO attempts to optimize scan step size (**a**), detector size (**b**), probe defocus (**c**), and aperture size (**d**).

The results from BO allowed us to estimate the entire 4D parameter space and observe how optimal experimental conditions depend on the total electron dose. As shown in Fig. 6, small probe and scan step size produce better results at extremely low-dose regimes. However, with increasing total electron counts, one can theoretically use a larger scan step size (> 5 Å) given sufficient probe overlap and detector pixels. Similarly, it is more advantageous to use a relatively small detector size (e.g. 128 × 128) at low dose levels as more pixels lead to poor SNR. Lastly, with the exception of 100 e$^-$/Å$^2$, most of the optimal conditions found by BO have large (> 30 mrad) aperture size, indicating that in addition to its size, the probe structure also influences the quality of ptychographic reconstructions. This agrees with previous literature^18^ that shows specialized focusing optics can produce superior images. Because the focusing probe is typically characterized by a few physical parameters in electron microscopy, we believe the probe structure can be further optimized using the BO framework.

## Discussion

As a general technique for black-box optimization, BO provides a framework that easily extends to other parameter tuning tasks beyond the eight reconstruction parameters and four experimental parameters studied in this work. For instance, in multi-slice ptychography, one can optimize model parameters such as the sample thickness and the number of layers. By minimizing the data error between reconstruction and data, BO facilitates automatic estimation of experimental conditions, including probe defocus or global scan position errors, which cannot be measured accurately by electron microscopes. In addition, our parameter tuning workflow has implications for non-iterative reconstruction techniques^47,48^ and other inverse problems such as X-ray ptychography and tomography.

Currently, automatic parameter tuning and typical electron ptychography experiments operate on similar time scales (a few hours), which prohibits online optimization. However, multiple hardware and software improvements can be made to further enhance computational efficiency. First of all, because the majority of the workflow is ptychographic reconstruction, utilizing more advanced hardware, such as high memory-bandwidth GPUs can significantly reduce the processing time by more than tenfold compared to the current work. The total processing time can be further shortened by carrying out multiple reconstructions in parallel and using multi-points optimization strategies^49^. In addition, experienced scientists may leverage additional properties about reconstruction algorithms or data to reduce the parameter space in BO. The reduction can be implemented at the beginning or during the parameter tuning workflow. Lastly, recent developments such as physics-informed BO^50^, causal BO^51^, and deep kernel learning^29^, may provide more solutions that facilitate more intelligent decision-making by exploiting underlying relationships between different parameters.

Lastly, we want to emphasize the importance of the objective function for optimizing reconstruction quality. An ideal objective function should reflect the accuracy of ptychographic reconstructions so that automatic parameter tuning produces true sample structures rather than artifacts. In numerical simulations, accuracy can be directly quantified since a ground truth is available. For experimental data, the complete information is unknown to researchers and many prevailing evaluation metrics (e.g. the FRC score) only characterize the precision of reconstructions. The FRC analysis is often used in ptychography literature^16,33,38,40,52^ and correlates with accuracy to some extent, especially when the dominating factor is dose (Supplementary Fig. 4). Nevertheless, there exist reconstruction and experimental parameters that lead to deceiving results with high precision but low accuracy. For instance, applying image regularization techniques such as de-noising may “improve” the FRC score by removing noisy artifacts in the object but reduce sharp features if the image is over-smoothed. The experimental data of bilayer MoSe$_2$/WS$_2$ provides another example as its 1-bit FRC resolution is close to 0.2 Å (Supplementary Table 2), while the spatial resolution estimated based on the diffractogram is only 0.42 Å (Fig. 2c). To avoid such systematic bias, one should be attentive to limitations of different metrics and, if possible, incorporate additional knowledge into the parameter tuning workflow to directly optimize accuracy, or combine with precision measurement via multi-objective optimization^53^.

## Conclusion

In summary, we demonstrated a human-out-of-loop parameter tuning framework for electron ptychography based on Bayesian optimization with Gaussian processes. The workflow does not require strong prior knowledge about the input data or advanced reconstruction techniques, and can automatically determine parameters that correctly account for various experimental errors and produce high-resolution ptychographic reconstruction of experimental data. The results suggest the most important parameters for the bilayer MoSe$_2$/WS$_2$ data are the number of probe modes and position correction, which are in good agreement with human experiences and theoretical studies. Similarly, BO can be used to search for the optimal experimental conditions in complex multi-dimensional parameter space, allowing better designs for ptychography applications such as low-dose imaging. With rapid developments in computing hardware, software, and advanced BO techniques, we anticipate that fully automatic parameter tuning will achieve sufficient throughput for real-time electron ptychography applications.

## Methods

### Ptychographic reconstruction

Ptychographic reconstructions were carried out using a customized library based upon the PtychoShelves package^34^. The library, which is maintained at https://github.com/yijiang1/fold_slice, supports electron ptychography data and provides a python interface. For reconstruction parameter tuning studies, we further modified the code to allow for time-limited reconstruction instead of standard iteration-limited reconstruction.

Supplementary Table 1 summarizes eight types of reconstruction parameters that are explored during automatic parameter tuning. These parameters influence both the quality and efficiency of ptychographic reconstruction and are frequently adjusted for different experimental data. The core algorithm is the maximum likelihood ptychography with a least-squares solver^33^, which provides both Gaussian and Poisson probability distribution to model data noise. The method also used a mini-batch update strategy to efficiently balance reconstruction speed and convergence rate. Thus, the number of diffraction patterns in each batch and the batch selection scheme (sparse vs. compact) are tunable parameters in reconstruction. In addition, the number of probe modes in mixed-states ptychography^35^ can be adjusted to account for partial coherence^16^ and probe vibration^54^. In the orthogonal probe relaxation (OPR) technique^36^, which is often used to reduce artifacts caused by probe variation in a single scan, the number of orthogonal modes kept in truncated singular value decomposition controls the amount of structural changes allowed at each scan position. Moreover, position correction can refine inaccurate scan positions and intensity correction accounts for changes in probe intensity. Lastly, the “multimodal” option specifies if all or only the first probe mode are used to update the object function.

In general, the upper bounds for the number of mixed-states probe modes, the OPR modes, and the batch size are limited by the data size and the GPU (NVIDIA GeForce GTX 1080 Ti) memory. For simplicity, we define position correction, intensity correction, and multimodal as binary variables. If an option is set to true, then the feature is used throughout the entire reconstruction process.

### Bayesian optimization with Gaussian process

Bayesian optimization was carried out with the Scikit Optimize library^55^. After each ptychographic reconstruction, the image quality and corresponding parameters are used to update the GP model. Here we used the Matern kernel^56^—a popular covariance function defined as:

1 $$\begin{aligned} k(x_{i},x_{j}) = \frac{1}{\Gamma (\nu )2^{\nu -1}}\left( \frac{\sqrt{2\nu }}{l}d(x_{i},x_{j}) \right) ^{\nu }K_{\nu }\left( \frac{\sqrt{2\nu }}{l}d(x_{i},x_{j})\right) \end{aligned}$$

where $d(x_{i},x_{j})$ measures the Euclidean distance between two points, $\Gamma (\nu )$ is the gamma function, and $K_{\nu }$ is the modified Bessel function of the second kind. $\nu$ is a positive parameter that controls the smoothness of the kernel and *l* is the length scale, which is updated during BO. For all reconstructions parameter tuning studies in this paper, $\nu$ is set to 1.

To sample the next point, we used a portfolio strategy known as “GP Hedge”^57^, which selects points using a pool of acquisition functions, including negative probability of improvement^58^, expected improvement^59^, and upper confidence bound^60^. Lastly, the automatic parameter tuning workflow randomly sample a small number of initial points for Gaussian Process modeling before the Bayesian optimization process.

### Experimental parameter tuning

For experimental parameter optimization, we generated a simulated potential of bilayer MoS$_2$ with a 30$^{\circ }$ twist. Single-atom potentials^61^ placed at appropriate coordinates were summed to generate the full potential of the bilayer. Interpolation was used to avoid the large singularity at the center of individual potentials. The resulting potential is 2048 × 2048 pixels with a pixel size of 0.125 Å.

For all simulated data, the scan field of view was about 6 nm × 6 nm and the pixel size was fixed at 0.125 Å. The beam energy was set 80 keV. Each 4D dataset was simulated assuming the strong phase approximation, and then reconstructed with a single probe mode, compact batch selection scheme, and no additional corrections. The batch size was chosen dynamically to fully utilize the GPU memory. All reconstructions were run for 500 iterations on a single NVIDIA V100 GPU, and took from $\sim$10 s to $\sim$10 min, depending on the data size.

As summarized in Supplementary Table 2, most experimental parameters are defined as continuous variables, giving an infinite parameter space. Bayesian optimization attempted to maximize the SSIM^46^ between a reconstruction and the ground truth. The Hammersley sampling method^62^ was used to explore 100 initial points that randomly cover the entire parameter, after which BO was used to search for additional 700 sets of parameters.

## Supplementary Information

Supplementary Information.

## Data Availability

The experimental electron ptychography data used for reconstruction parameter tuning is published in ref.^16^. An example script of Bayesian optimization is available at https://github.com/yijiang1/fold_slice. All other data and code are available from the corresponding author at reasonable request.
