# Supplementary material for: Automatic parameter selection for electron ptychography via Bayesian optimization
Source: Sci Rep. 2022 Jul 19;12:12284. doi: 10.1038/s41598-022-16041-5 (PMC9296498; doi:10.1038/s41598-022-16041-5)
Supplement: Supplementary file 1 — Supplementary Information. [file 41598_2022_16041_MOESM1_ESM.pdf]

Supplementary Information

Michael C. Cao[1], Zhen Chen[2], Yi Jiang[3*], and Yimo Han[1*]

[1]Department of Materials Science and NanoEngineering, Rice University, Houston, TX, USA 77005
[2]School of Materials Science and Engineering, Tsinghua University, Beijing 100084, China
[3]Advanced Photon Source, Argonne National Laboratory, Lemont, IL, USA 60439

May 27, 2022

1 Figures

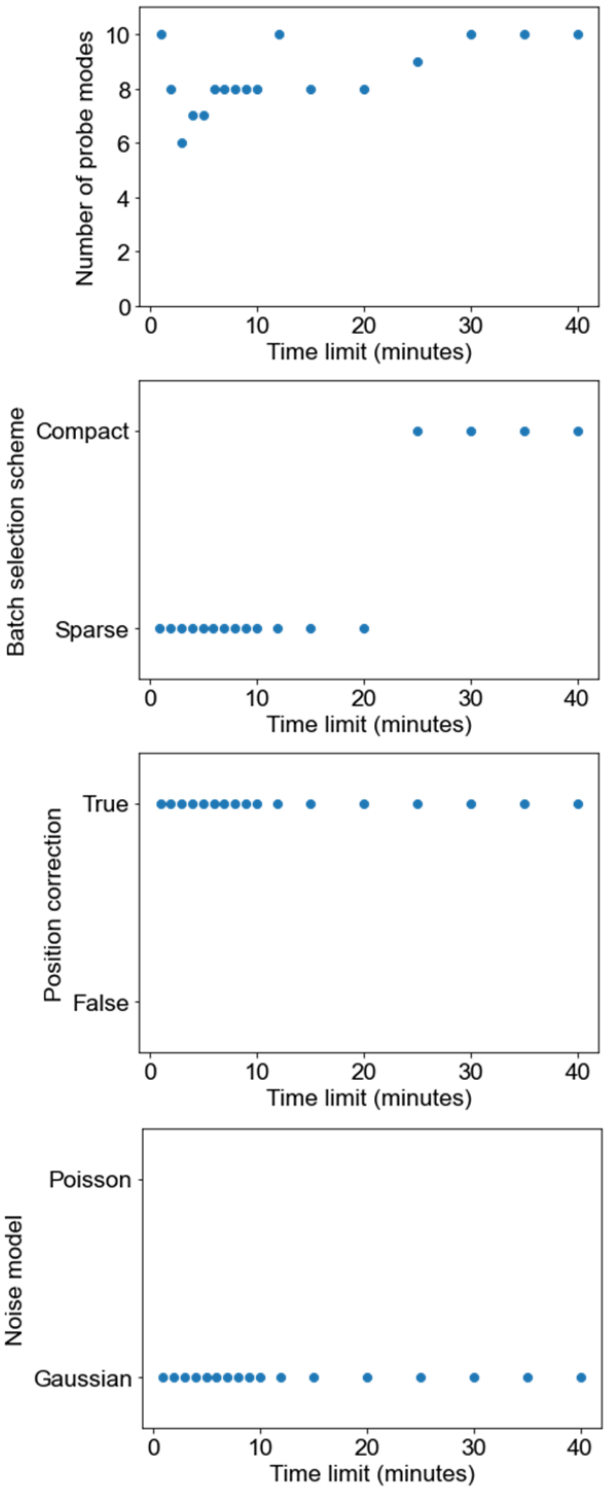

Figure S1: Optimal reconstruction parameters of an experimental dataset of bilayer $MoSe_2/WS_2$. At each time limit, eight different types of parameters were optimized by automatic parameter tuning with Bayesian optimization.

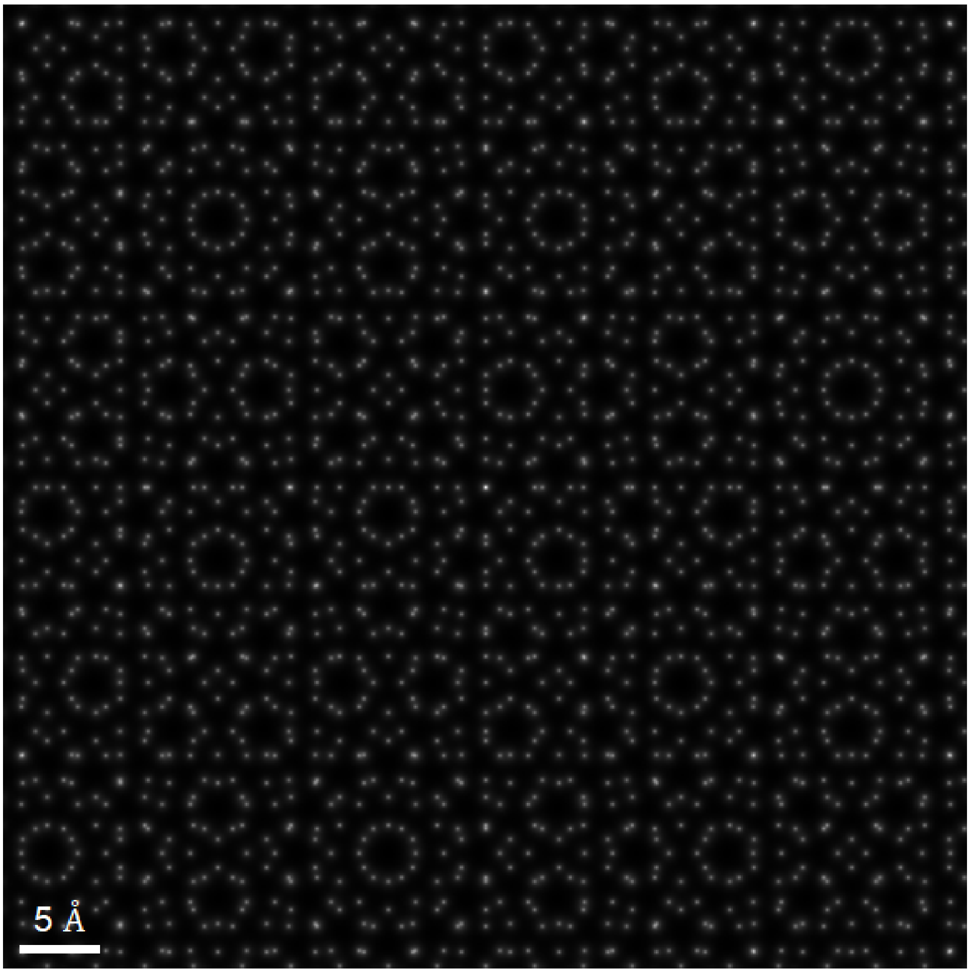

Figure S2: Projected potentials of simulated bilayer MoS_2 structure with a 30° twist. The object was used to simulate electron ptychograpy data in automatic experimental parameter optimization.

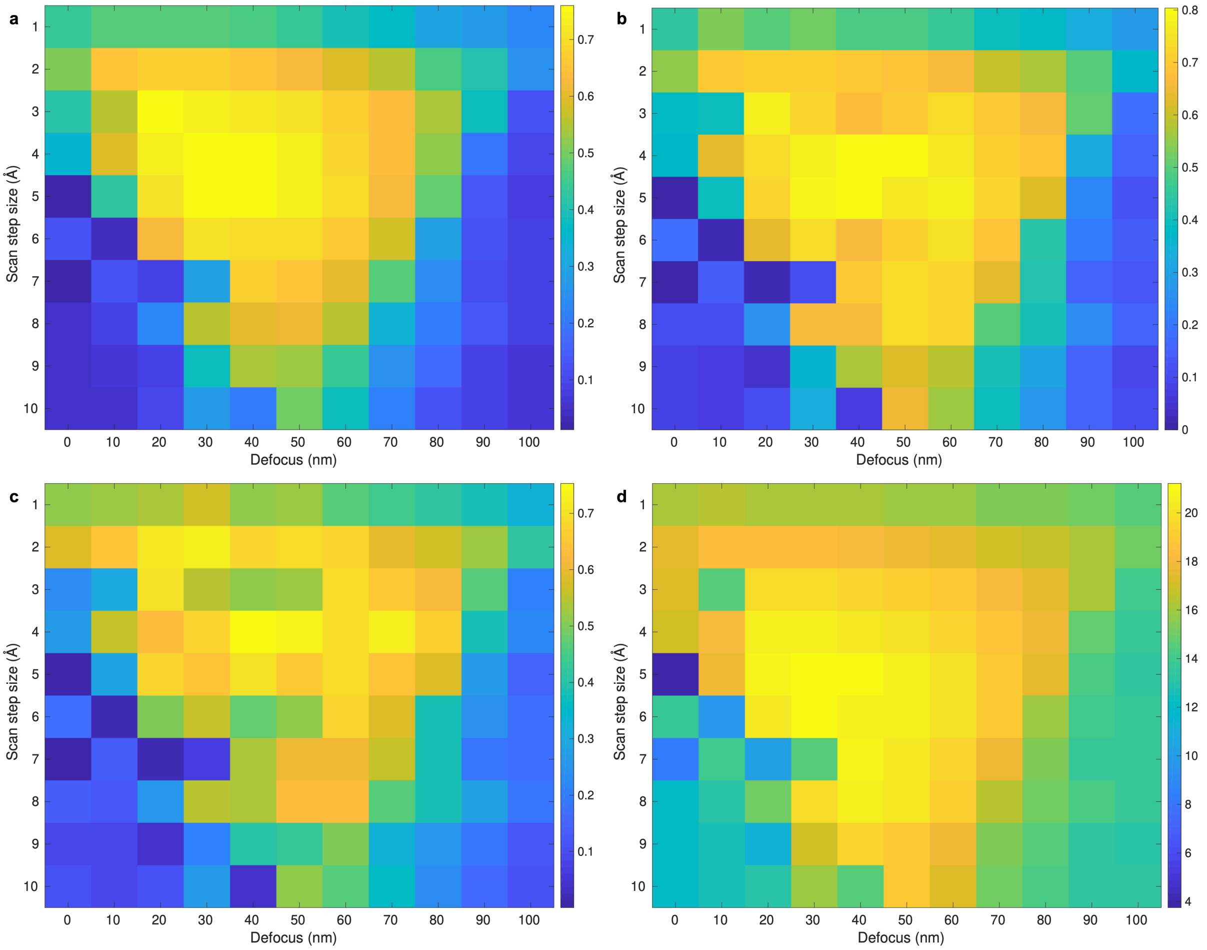

Figure S3: Comparison of metrics for evaluating ptychographic reconstructions of simulated bilayer MoS_2 structure. Diffraction patterns with various scan step sizes and probe defocus were generated following the descriptions in the Method section. The detector size, aperture size, and total electron dose were set to 256 × 256, 20 mrad, and 10000 $e^-/Å^2$, respectively. For each point in the 2D parameter space, the accuracy of corresponding reconstruction was evaluated by (a-c) Structural similarity index measure with a window size of 1.5, 5, and 10, respectively and (d) Peak signal-to-noise ratio.

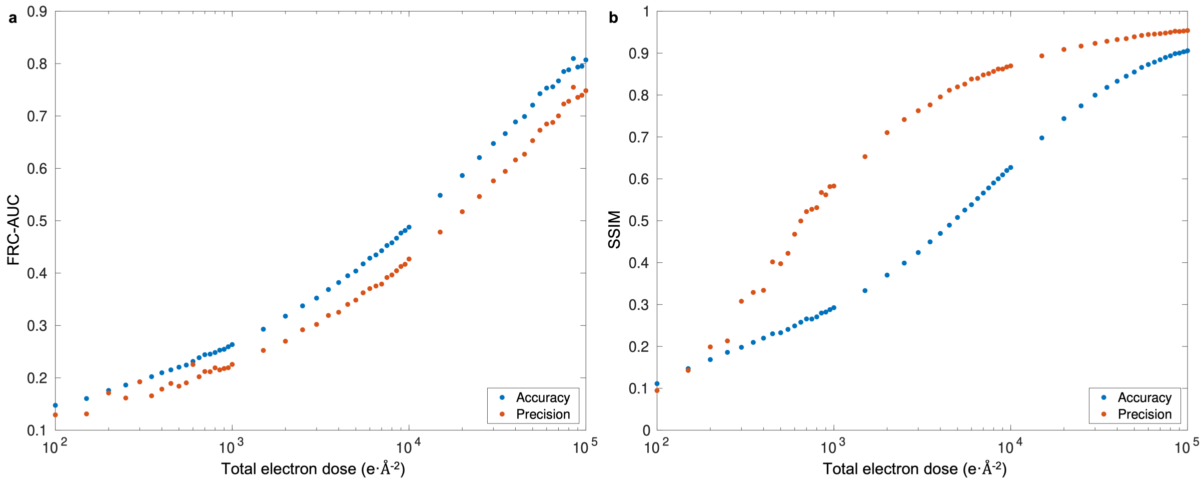

Figure S4: Precision and accuracy of ptychographic reconstructions of simulated bilayer MoS_2 structure. At each dose level, two independent datasets were simulated assuming 2 Å scan step size, 50 nm probe defocus, 20 mrad aperture size, and 256 $\times$ 256 detector size. Precision (similarity between two ptychographic reconstructions) and accuracy (averaged error between reconstructions and the ground truth) were calculated by (a) the area under the FRC curve and (b) Structural similarity index measure.

2 Tables

Reconstruction Parameter	Value
Number of mixed-state modes	1, 2, 3, 4, 5, 6, 7, 8, 9, 10
Batch size	60, 120, 180, 240, 300
Batch selection scheme	Sparse, Compact
Number of OPR modes	0, 1, 2
Intensity correction	True, False
Position correction	True, False
Modal update	True, False
Noise model	Gaussian, Poisson

Table 1: Reconstruction parameters and possible values in automatic parameter tuning. There are a total number of 4800 possible combinations.

Figure	1-a,b	1-c,d	1-e,f	1-g,h
Reconstruction parameters				
Number of mixed-state probe modes	7	7	7	**1**
Batch size	300	**60**	300	300
Batch selection scheme	Sparse	Sparse	Sparse	Sparse
Number of OPR modes	0	0	0	0
Intensity correction	False	False	False	False
Position correction	True	True	**False**	True
Noise model	Gaussian	Gaussian	Gaussian	Gaussian
Modal update	False	False	False	False
Reconstruction quality				
Area under the FRC curve	0.818	0.709	0.601	0.239
1-bit FRC resolution (Å)	0.206	0.433	0.438	0.997
SSIM	0.896	0.816	0.791	0.518

Table 2: Reconstruction parameters and quality evaluations of bilayer $MoSe_2/WS_2$ sample. The parameters correspond to the reconstructions that are shown in Figure 3. The automatic parameter tuning with BO was used to optimize the area under the FRC curve, which reflects the similarity between two independent reconstructions. This is consistent with visual inspection and other metrics such as the 1-bit FRC resolution and SSIM.

Experimental parameter	Range
Scan step size (Å)	1.5 to 15
Aperture size (mrad)	5 to 35
Probe defocus (nm)	0 to 1000
Detector size (# of pixels)	64×64, 128×128, 196×196, ..., 1024×1024

Table 3: Experimental parameters and ranges in automatic parameter optimization for dose-limited electron ptychography.
